# Prolonged life of human acute hippocampal slices from temporal lobe epilepsy surgery

**DOI:** 10.1038/s41598-018-22554-9

**Published:** 2018-03-07

**Authors:** J. Wickham, N. G. Brödjegård, R. Vighagen, L. H. Pinborg, J. Bengzon, D. P. D. Woldbye, M. Kokaia, M. Andersson

**Affiliations:** 1 Epilepsy Centre, Department of Clinical Sciences, Lund University, Sölvegatan 17, 223 62 Lund, Sweden; 2 Epilepsy Clinic and Neurobiology Research Unit, Department of Neurology, Copenhagen University Hospital, Rigshospitalet, Building 6931, Blegdamsvej 9, DK-2100 Copenhagen, Denmark; 3 Department of Clinical Sciences, Lund University, Skåne University Hospital Lund, S-22185 Lund, Sweden; 4 Lund Stem Cell Center, BMC B10, Klinikgatan 26, 221 84 Lund, Sweden; 5 Laboratory of Neuroplasticity, Center for Neuroscience, University of Copenhagen, Copenhagen, Denmark

## Abstract

Resected hippocampal tissue from patients with drug-resistant epilepsy presents a unique possibility to test novel treatment strategies directly in target tissue. The post-resection time for testing and analysis however is normally limited. Acute tissue slices allow for electrophysiological recordings typically up to 12 hours. To enable longer time to test novel treatment strategies such as, e.g., gene-therapy, we developed a method for keeping acute human brain slices viable over a longer period. Our protocol keeps neurons viable well up to 48 hours. Using a dual-flow chamber, which allows for microscopic visualisation of individual neurons with a submerged objective for whole-cell patch-clamp recordings, we report stable electrophysiological properties, such as action potential amplitude and threshold during this time. We also demonstrate that epileptiform activity, monitored by individual dentate granule whole-cell recordings, can be consistently induced in these slices, underlying the usefulness of this methodology for testing and/or validating novel treatment strategies for epilepsy.

## Introduction

Epilepsy is a multifactorial neurological disease characterised by pathological hyper-synchronised activity of neurons manifested as recurrent spontaneous seizures. In the majority of cases, symptomatic treatment by currently available anti-epileptic drugs (AEDs) suppresses seizures. However, in about 30–40% of patients, AEDs are ineffective at controlling seizures, leaving patients with diminished quality of life^1^. In these patients, a treatment option is resection of epileptic tissue, provided the seizure-generating focal area is reliably identified and is located outside eloquent cortex^2^. One of the most common structures for focal seizure origin is the temporal lobe, where resections constitute an effective and relatively low risk treatment for a defined patient population^2^. This therapeutic surgical procedure also provides a unique opportunity for pathophysiological evaluation of human epileptic tissue, including the possibility to maintain tissue as live slices readily accessible for electrophysiological recordings^3,4^. Slice preparations from resected tissue are extremely valuable not only for providing information on pathological network mechanisms of the epileptic brain tissue but also for validating novel treatment strategies developed in preclinical studies using animal models^4–8^. Such a validation step is of particular importance considering that the resected human epileptic tissue is drug-resistant and can give indication if new treatments will be effective against refractory seizures^6,9^. Moreover, at present there are few, if any, drug-resistant animal models of epilepsy, and those that have been developed are extremely time and labour consuming^10^.

A number of research groups have studied acute human epileptic tissue slices with epileptic activity induced by various chemical manipulations, such as [0Mg^2+^], high [K^+^] and 4-aminopyridine (4-AP)^7,11,12^. These studies utilised an interface-recording chamber to reliably obtain epileptiform activity^12^. It is generally accepted that the interface-recording chamber provides higher oxygen-levels in the slices, compared to a submerged chamber, and therefore enables neurons to fire action potentials (APs) at higher frequencies for a prolonged period of time, allowing for the generation of seizure-like events (SLE)^12–14^. A major drawback of the interface-recording chamber, however, is that it precludes the visual guidance of a pipette for patch-clamp recording of individual cells in the slices, since water-submerged 20× or 40× objectives cannot be used in the microscope. An attractive alternative to the interface-recording chamber could be the dual-flow system^15^ designed to maximise oxygen levels by providing a laminar flow of oxygenated artificial cerebral spinal fluid (aCSF) on both sides of the submerged brain slice. This system has been reported to enable recordings of sustained epileptiform activity in acute rodent hippocampal slices, with the advantage of allowing visually guided whole-cell patch-clamp recordings^15,16^. However, this approach has not yet been applied to human brain slices^12^. The objective of the present study was two-fold: (i) to test the hypothesis that incubation time for human brain slices, using the interface incubation can be extended to 48 h without significantly compromising slice quality; and (ii) to establish a dual-flow submerged chamber system, enabling visually guided whole-cell recordings during epileptiform activity in human brain slices. The first objective was motivated by the need to increase yield of data from each occasion when human brain tissue becomes available, as well as to extend the time for allowing viral vector expression and thereby the validation step for gene expression and/or therapy effectiveness in human pharmacoresistant epileptic tissue.

## Material and Methods

Temporal lobe resection tissue blocks were obtained after surgery from 16 patients with pharmacoresistant temporal lobe epilepsy (seizures for 5 to 35 years and ages 6 to 57 years) from Copenhagen University Hospital and Lund University Hospital (see Table 1. for patient table). Post-surgicals evaluation of the hippocampus were performed by a pathologist at respective hospitals, and diagnosis for hippocampal sclerosis was determined according to ILAE guidelines^17^. The use of resected patient tissue and following procedures were approved by the local Ethical Committee in Copenhagen (H-2-2011-104) and Lund (#212/2007) and were performed in accordance with the Declaration of Helsinki. Written informed consent was obtained from all subjects prior to each surgery.

**Table 1:** Patient data.

	Patient	Resistance to >2AEDs	Age at surgery (yrs)	Duration of epilepsy (from onset; yrs)	Seizure frequency (n/mo)	AEDs at surgery	Hippocampal pathology
*CPH*	1	Yes	52	25	1 (KFA)	LTG, LEV	HS
*CPH*	2	Yes	54	14	5 (KFA)	LEV, LTG	HS
*CPH*	3	Yes	41	34	8 (SFA), 5 (KFA)	ZNS, LAC, CLB	HS
*Lund*	4	Yes	36	35	5	LTG, VPA	HS
*CPH*	5	Yes	55	19	8 (SFA), 4 (KFA)	LEV, LAC	HS
*CPH*	6	Yes	31	12	1 (SFA), 1 (KFA)	LTG, LEV	HS
*CPH*	7	Yes	57	5	9 (KFA)	OXC	HS
*CPH*	8	Yes	34	8	3 (KFA)	CBZ, LEV, LAC	HS
*Lund*	9	Yes	28	16	3	LTG, LEV	*Normal*
*CPH*	10	Yes	18	12	8 (SFA), 8 (KFA)	VPA	HS
*CPH*	12	Yes	44	16	5	BV, ZNS, LTG	*Normal*
*CPH*	13	Yes	19	13	6 (SFA) 6 (KFA)	LEV, CBZ	HS
*Lund*	14	Yes	23	22	4 (KFA)	LEV, LAC	HS
*CPH*	15	Yes	36	26	12 (SFA), 12 (KFA)	LTG, LEV	HS
Lund	16	Yes	6	5	30	LTG, LEV	HS

Seizure frequency reported by patients in Copenhagen (CPH) reported as simple focal seizures (SFS) and complex focal seizures (CFS). If SFS are not noted the patient does not report experiencing auras/SFS. Abbreviations as follows carbamazepine (CBZ), clobazam (CLB), Brivaracetam (BV), lacosamide (LAC), lamotrigine (LTG), levetiracetam (LEV), oxcarbazepine (OXC), valproate (VPA) and zonesamide (ZNS) and hippocampal sclerosis abbreviated as (HS).

### Acute slice preparation

The hippocampal tissue is surgically removed *en bloc* and then cut in the coronal plane once or twice to establish orientation of the hippocampal structures and aid in the positioning of the tissue when slicing. The resected tissue is then placed in ice-cold sucrose solution, frozen to slush, containing (in mM): 200 sucrose, 21 NaHCO3, 10 glucose, 3 KCl, 1.25 NaH2PO4, 1.6 CaCl2, 2 MgCl2, 2 MgSO4 (all from Sigma-Aldrich, Sweden), adjusted to 300–310 mOsm, 7.4 pH. The tissue is either transported from Copenhagen University Hospital, Rigshospitalet to Lund (60–90 min) or between the surgery room at Lund University Hospital and the electrophysiology lab in the neighbouring building (15 min). Depending on the orientation of the hippocampus, the tissue is either trimmed to give a better surface to glue on, or glued straight away onto the cutting platform. The 400 µm thick, coronal slices are cut with a vibratome (VT1200, Leica Microsystems) in ice-cold sucrose solution, continuously bubbled with 95% O_2_ and 5% CO_2_.

### Acute slice incubation

**Figure 1:**
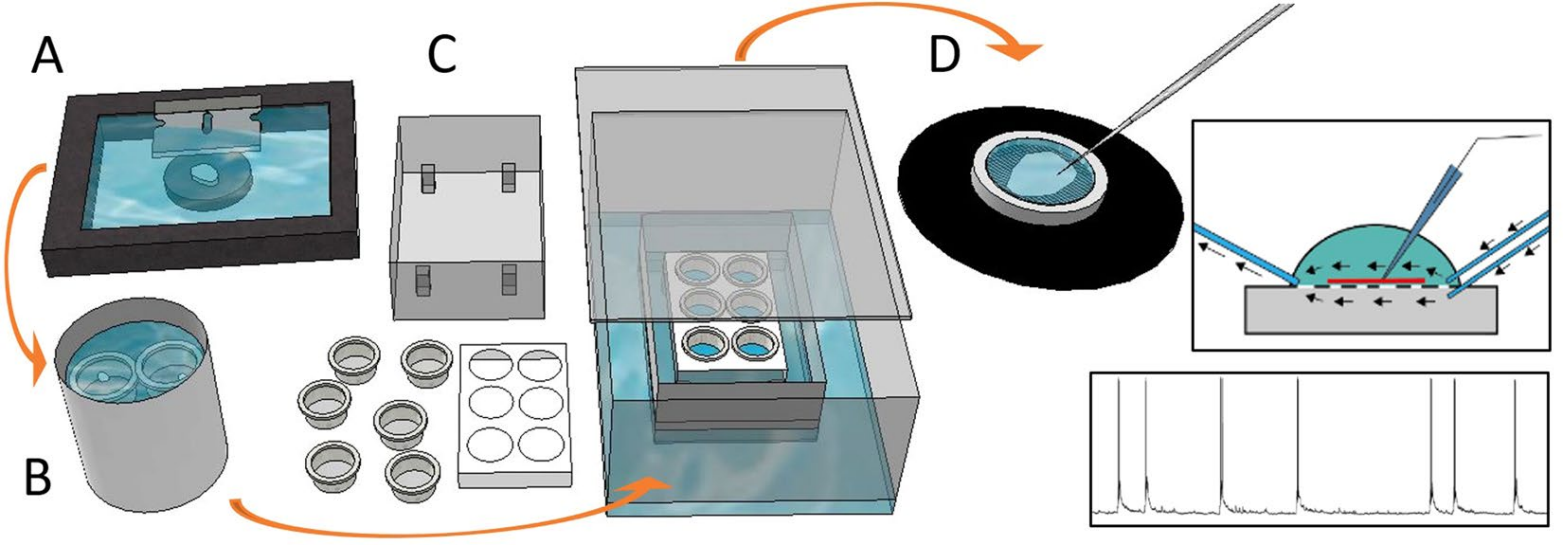
Overview of Experimental setup. (**A**) The tissue is resected *en bloc* and sliced on a vibratome. (**B**) The slices rest for 15 min submerged in continuously bubbled (95% O_2_/5% CO_2_) aCSF and (**C**) are then moved to the incubation chamber for 3–48 hours of incubation with a constant flow of bubbled aCSF in the small chamber and humidified air crated by bubbling the aCSF in the outer box which is sealed with a lid. After incubation the slices are transferred to the dual-flow recording chamber (**D**) where electrophysiological whole-cell patch-clamp recordings were made.

Slices were collected in a pre-incubation bath with aCSF, containing (in mM): 129 NaCl, 21 NaHCO_3_, 10 glucose, 3 KCl, 1.25 NaH_2_PO_4_, 2 MgSO_4_, and 1.6 CaCl_2_, adjusted to 300–310 mOsm, 7.4 pH, heated to 34 °C and continuously bubbled with carbogen (95% O_2_ and 5% CO_2_). Slices rested on nets, fully submerged, for 15–30 min before they were transferred to the interface incubation chamber. This incubation consisted of a closed chamber with humidified air inside created by a bubble stone continuously bubbling carbogen into aCSF covering the bottom of the closed chamber. Inside this chamber, an open box with a constant flow of carbogenated aCSF was placed containing the slice holder, with six holes for cell culture insets (Millipore, Germany), positioned in the box to just touch the surface of the circulating aCSF (Fig. 1). The slices were placed onto the cell culture membranes of the insets, resulting in constant aCSF flow below the membrane and humidified air from above. The membrane enabled slices to access aCSF (with nutrients and defined ion composition) while the humidified air gave maximised access to oxygen, preventing drying of slices. Care was taken to minimize bacterial growth, especially for slices incubated for 48 hours, and the aCSF in circulation was changed every 12 hours to prevent accumulation of bacteria. During the course of experiments, a UV-C light was added to the circulation tubing to eliminate bacteria in the aCSF. It was installed in the recirculation loop of the aCSF, outside of the incubation box, and great care was taken to not illuminate anything else except the circulating aCSF. The slices rested in the interface chamber, at room temperature between 3 and 48 hours.

The study was based predominantly on the analysis of both structural and functional characterization of dentate gyrus of the hippocampus, since this was the area most commonly identified to be less damaged and present in all tissue pieces that we received from the surgical resections of the temporal lobe.

### Electrophysiology

Electrophysiological recordings were performed with glass capillary electrodes (tip resistance between 2.5 and 6 MΩ) backfilled with a solution containing in mM: 122.5 K-gluconate, 12.5 KCl, 10 KOH-HEPES, 0.2 KOH-EGTA, 2 Mg-ATP, 0.3 Na3GTP, and 8 NaCl, pH 7.2–7.4 (mOsm 290–300) with HEKA amplifier (HEKA, Germany) controlled with HEKA Patchmaster software. Visualisation of the cells was achieved by an Olympus microscope BX51WI (Olympus, Germany). The slices were individually transferred to the dual flow-recording chamber and held in place by a horseshoe-shaped piece of platinum wire. The slice was perfused with carbogenated aCSF, preheated to 32 °C, at a flow rate of 2 ml/min. The slice rested on a metal grid allowing for a laminar flow of aCSF directed above and beneath the slice, ensuring a high amount of oxygen available for the slice and enabling the use of a submerged objective for infrared differential contrast (IR-DIC)-imaging, giving visual cues for patch-clamp recordings. Visual cues for whole-cell patch clamp excluded neurons with a dark distinguishable nucleus or a swollen or shrunken soma. After successful giga-seal formation, the patch was ruptured and the resting membrane potential (RMP) was immediately measured in current-clamp recording mode before continuing with other recording sequences. Experiments with an access resistance over 30 MΩ were excluded from analysis. For field-recordings, capillaries with a tip resistance between 1 and 3 MΩ, backfilled with aCSF were used.

### Incubation and induction of epileptiform activity

Slices were transferred for recordings, starting after 3 hours to allow for recovery of dendritic spines that retract in the ice-cold cutting solution, as reported in preparations of acute rodent hippocampal slices^5^. Electrophysiology recordings were obtained from slices incubated from 3 to 48 hours. The initial RMP-measurement was followed by depolarising current steps (stepwise increasing with 50 pA/step) to determine AP-threshold and firing pattern. Spontaneous activity of neurons was recorded in current-clamp mode for at least 15 min. Two different types of excitability-enhancing aCSF-solutions were used, either [0Mg^2+^]-aCSF alone, with magnesium omitted, or [0Mg^2+^]-aCSF containing the potassium-channel blocker 4-AP (100 µM). Following recording, the slices were fixed in 4% paraformaldehyde in phosphate buffered saline (PBS) overnight and stored submerged in Walter’s antifreeze solution (ethylene glycol and glycerol in PBS) at −20 °C.

Immunohistochemistry To produce tissue samples suitable for immunohistochemical staining, slices were removed from the antifreeze solution and washed three times in KPBS, embedded in a solution, containing 300 g/L egg-albumin (Sigma) and 30 g/L gelatin (Sigma) in Milli-Q water, and stored at −20 °C. Further sectioning was performed using a cryostat (Cellab Nordia AB) to produce 20 µm coronal sections. These were mounted on slides (+charged Menzel-Glas, Thermo Scientific) and stored at −18 °C before staining. Slices were rinsed three times with KPBS and pre-incubated for 1 hour at room temperature in blocking solution, consisting of 10% normal donkey serum (NDS) in 0.25% Triton X-100 in KPBS (T-KPBS). After blocking, slices were incubated in darkness at 4 °C with the appropriate dilution of primary antibody in 5% NDS in T-KPBS (see Suppl. Table 1) overnight. After rinsing three times in T-KPBS, they were incubated for 2 hours in darkness at room temperature with secondary antibody (1:200) in 5% NDS in T-KPBS (see Suppl. Table 1), after which they were rinsed three times in T-KPBS. The procedure was then repeated for the second staining. After the secondary antibody was applied, the slides were rinsed once in T-KPBS and twice in KPBS. Finally, slides were coverslipped with DABCO (Sigma-Aldrich D2522). In the Iba1 + GFAP-stainings, Hoechst 33342 (Hoechst AG) diluted to 1:1000 in DABCO was applied. Images were taken using an Olympus BX61 microscope (Olympus, Germany) fitted with a CCD camera connected to a Windows PC with cellSens Dimension software (Olympus, Germany). For NeuN + CASP3- and GFAP + Iba1-stainings, images overviewing the dentate gyrus and an area surrounding the tip of the dentate gyrus were taken, respectively, at 20× magnification. For NeuN + NPY-stainings, images were taken overviewing the dentate gyrus and the hilus at 10× magnification.

### Statistics and Analysis

Analysis of electrophysiology recordings was performed with IGOR Pro (Version 6.3, Wavemetrics) and Mini analysis software (Synaptosoft). Postsynaptic potentials (PSPs) were automatically detected with a detection threshold of 1 mV (Synaptosoft) and subsequently checked manually to eliminate double peaks during a 60 s timeframe. To further analyse the PSP properties, the first 12 PSPs from the five cells with the highest PSP frequency in each group were added together resulting in 60 PSPs from each of the time points: 3, 24 and 48 hours respectively. The parameters of PSPs and APs were detected and analysed automatically by Mini analysis software (Synaptosoft), with the duration measured as the width at half-amplitude. The resting membrane potential was calculated by an average of data points from the first 100 ms from current clamp recording start. The distribution of data was tested with D’Agostino & Pearson normality test as well as plotted according to frequency in histogram to evaluate if the data were normally distributed before further statistical analysis in Prism software (Graphpad 7). Normally distributed data were analysed with one-way ANOVA, while for data detected as not normally distributed, the Kruskal-Wallis test was used (Prism software, Graphpad 7). Analyses of immunohistochemical data were done using the Fiji/ImageJ software (https://imagej.nih.gov/). The area of the dentate gyrus was measured as the area between the innermost and outermost granule cells along the entire length of the structure, from which NeuN-positive and CASP3-positive cells were individually counted. Values for NeuN-positive cells/mm^2^, CASP3-positive cells/mm^2^ and CASP3-positive cells/NeuN-positive cells were calculated for each slice. NPY-positive cells/mm^2^ were calculated from NPY-positive interneurons in the hilus. The hilus was delineated by the border between granular cell layer and the hilus and a straight line drawn between the end-points of dentate granule cell layer against CA3/4 area. Iba1-assessment was performed in an hilus area, adjacent to the tip of the dentate gyrus, measuring 5mm^2^, in which all cells were counted and assessed by as Ramified (inactive; small soma with fine cellular processes), Intermediate (bigger, elongated soma with thicker proximal processes) or Activated (round or amoeboid cells with few or short processes) microglia. Cells that could not be assessed as a specific state were also included when counting overall cell density. Values were calculated for Ramified cells, Intermediate cells, Activated cells, as well as total Iba1-positive cells/mm^2^. GFAP was assessed with optical densiometry (Image J^18,19^) using an area of 1mm^2^ in the hilus adjacent to the tip of the dentate gyrus, extracting values for minimum, maximum and mean grey values and integrated optical density. Statistical analyses were performed using SPSS Statistics 24 (IBM) and data from the 3, 24 and 48-hour time points in each patient were normalized against 0 h. Normality of data was examined using Shapiro-Wilk’s test of normality with normally-distributed data evaluated using repeated-measures ANOVA with post-hoc Bonferroni correction and non-normally distributed data assessed using Friedman’s test.

## Results

### No detectable changes in neuronal morphology or apoptosis in slices after 48 hours of incubation

**Figure 2:**
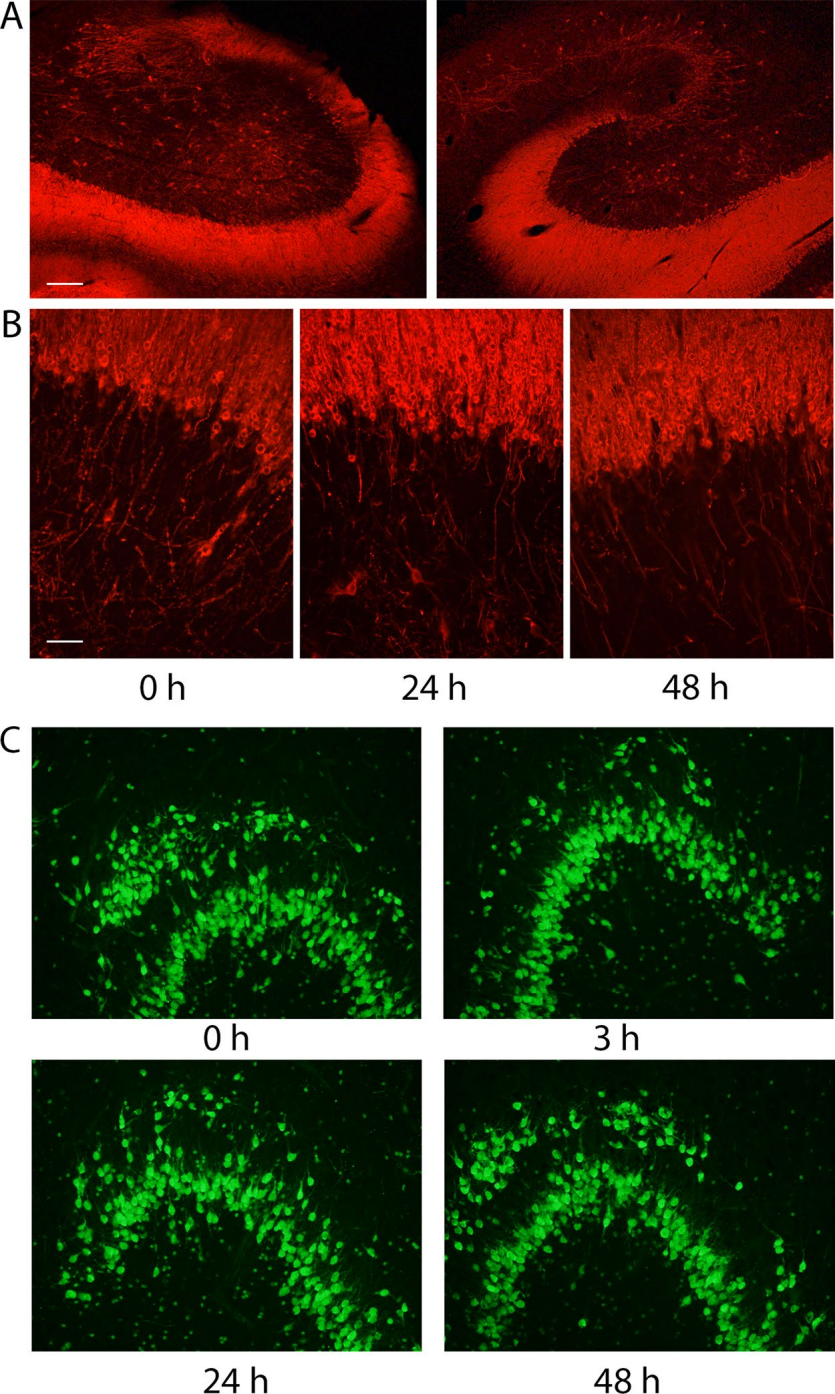
Interface incubation of human hippocampus maintain hippocampal organisation. (**A**) Hippocampal slices incubated for 0 and 48 hours from the same patient (patient number 12, no hippocampal sclerosis) stained for MAP-2 in red did not show any structural changes between time-points, (**B**) neither did the granule cells in the dentate gyrus differ between 0, 24 and 48 h from the same patient. (**C**) Staining for neuronal nuclear marker, NeuN in green, did not show any morphological differences between time-points in a patient diagnosed with hippocampal sclerosis (patient number 15). Scale bars 20 µm.

**Figure 3:**
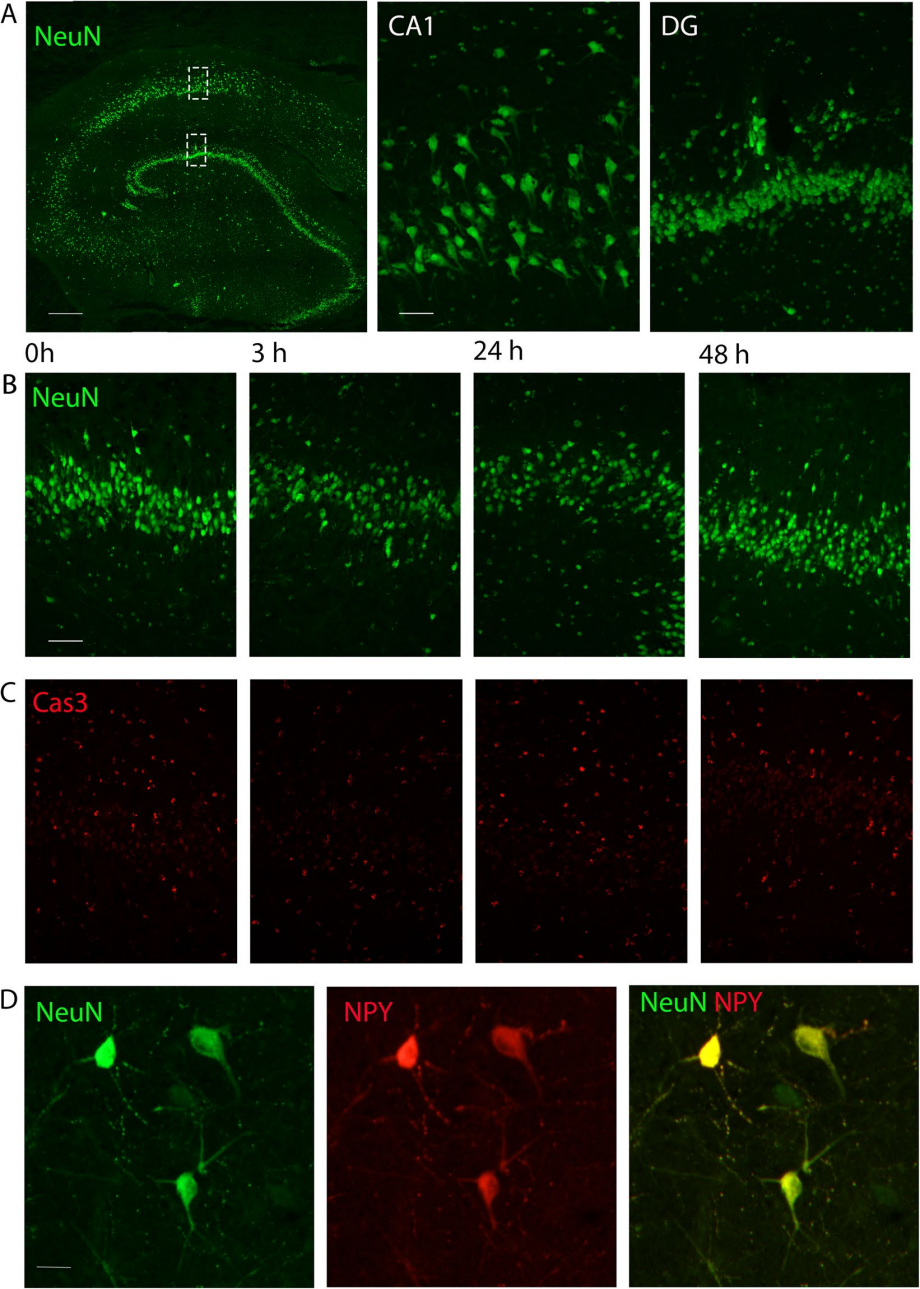
Human hippocampus shows no difference in the number of neurons, expression of apoptotic marker or NPY-interneuron number in interface incubation. (**A**) Neuronal nuclear marker, NeuN in green (scalebar 500 μm), outlining cell layers magnified to the right for CA1 and dentate gyrus (scalebar 100 μm). (**B**) No differences were found in number of NeuN-positive neurons in the dentate granular layer over the time studied (green, scalebar 100 μm, Patient 16), (**C**) nor in the number of neurons positive for the apoptotic marker Cas3 (red). (**D**) Example of NPY-expressing interneurons positive for NeuN (green) and NPY (red) found in the hilar region of dentate gyrus from 48 h time-point. Scalebar 20 μm.

The viability of human hippocampal slices incubated in our interface system was first examined by assessing several immunohistochemical markers at 0, 3, 24 and 48 hours. We chose to focus our entire analysis on dentate gyrus because it was always present and most preserved part of the hippocampus after the resection. Microtubule-associated protein 2 (MAP2) immunostaining showed clear and abundant neuronal morphologies in slices from both 0 and the 48-hour time point (Fig. 2A). A closer examination of the dentate granule cell layer did not reveal any differences in neuronal morphology between slices incubated for 0, 3, 24 or 48 h (Fig. 2B). To evaluate potential changes in neuronal density over time, we stained for the neuronal nuclear marker, NeuN, and counted number of cells in the granule cell layer of the dentate gyrus. Cell density analysis of NeuN-positive granule cells showed variability between patients but no signs of significant changes over time in any of the tissue obtained from individual patients (Fig. 3B and Supplementary Table 2). Consistent with maintained morphological features, counterstaining for apoptotic marker Cas3, did not show any increase in expression across the time-points studied (Fig. 3C and Supplementary Table 2). We also found the same number of cells positive for both neuropeptide-Y (NPY) and NeuN throughout the incubation, indicating that also NPY-expressing interneurons were viable over time (Fig. 3D and Supplementary Fig. S1 and Supplementary Table 2). Finally, microglial- and astrocytic activation was assessed in the slices. Microglial cells, labelled by Iba1 were counted and different cellular states (Ramified, Intermediate and Activated, respectively) were analysed in a 5 mm^2^ area in the hilus, adjacent to the tip of the dentate gyrus. A clear decrease in Ramified microglial cell numbers was observed from 0 h to 3 hours and further to 24 hours (45 ± 3.1 to 4.75, *n* = 3, p = 0.0131, paired *t-test*). Optical density measurements for GFAP-immunostaining within an area of 1 mm^2^ at the tip of the hilus of the dentate gyrus did not show any trend for change over the time of incubation (Supplementary Table S2) in any of the parameters measured. Taken together, these results suggest that there are neither detectable changes in morphology or overall density of neurons, nor astrocytes in the slices from different time points, although microglia activation increase over time in incubation.

### Intrinsic neuronal properties after 3, 24 and 48 hours of incubation

**Figure 4:**
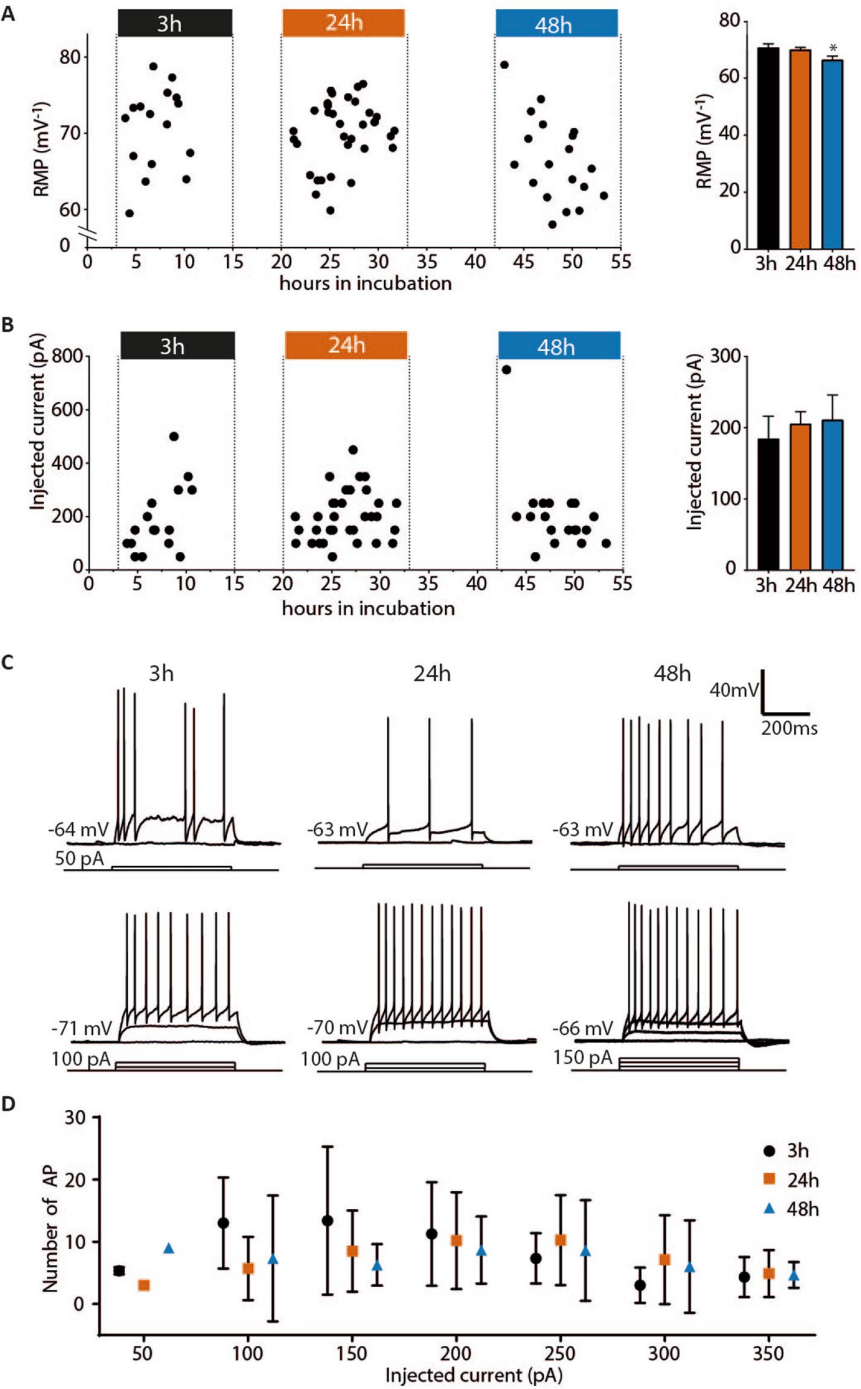
Intrinsic properties of human dentate granular cells. (**A**) The left graph show each cell, represented as a dot, with RMP on the y-axis and number of hours in incubation on the x-axis. Dotted lines and coloured blocks indicate the three time-groups (3 h, 24 h and 48 h). A small but significant change was detected in the RMP for the 48 hour group (*n* = 69, p = 0.0219, ANOVA) illustrated in the bar graph to the right (Tukey’s multiple comparison test: 3 hours, 70.62 ± 1.36 mV, *n* = 16, compared to 48 hours, 66.47 ± 1.27 mV, *n* = 19, p = 0,0414, 24 hours, 70.04 ± 0.74 mV, *n* = 34 compared to 48 h, 66.47 ± 1.27 mV, *n* = 19, p = 0.0372). No statistically significant differences between the 3 hour and 24 hour groups were detected (Tukey’s multiple comparison test: 3 hours, 70.62 ± 1.36 mV, *n* = 16, compared to 24 hours, 70.04 ± 0.74 mV, *n* = 34, p = 0.9198). (**B**) The left graph show the lowest current injection step (50 pA/step) needed to generate AP for each cell, represented by a dot, with amount of current injected on the y-axis and number of hours in incubation on the x-axis. The right graph illustrate the lowest current injection step needed to generate AP for cells binned to 3, 24 and 48 hours, with no differences between the three groups (*n* = 69, Kruskal-Wallis test, p = 0.5481). For Input resistance, AP threshold, AP amplitude and AP half-width with each cell represented by a dot and number of hours in incubation on the x-axis see Supplementary Fig. S1. (**C**), upper panel, example recordings of the lowest current injection step (50 pA/step) needed to generate AP in cells incubated for 3, 24 and 48 hours. Lower panel, example recordings of the highest number of AP generated during a depolarisation step from cells incubated for 3, 24 and 48 hours. (**D**) Number of AP plotted at each depolarisation step (50 pA/step) from 50 to 350 pA with mean number of AP and SEM for each group (3, 24 and 48 hours).

As neuronal density and overall morphology of neurons seemed to be unchanged after 48 h of incubation, we proceeded to confirm these morphological findings by assessing functionality of the neurons, starting by investigating their intrinsic properties at three time-points of the incubation period. Whole-cell patch-clamp recordings from dentate granule cells in the double-flow chamber were performed after interface chamber incubation for 3, 24 or 48 hours. The total number of granule cells recorded at the different time intervals were 16, 34 and 19 for 3, 24 and 48 hours, respectively. The RMP of all cells were estimated immediately after breaking the membrane for whole-cell recordings. A small but statistically significant reduction in average RMP in the 48 h group was detected (Fig. 4A, Table 2). The average RMP at 48 hours was 66.47 ± 1.27 mV, while at 3 and 24 hours RMP was 70.62 ± 1.36 and 70.04 ± 0.74 mV, respectively. Tukey’s test of multiple comparisons (*n* = 69, p = 0.0219, ANOVA) identified a significant difference between the 48 hours group and the 3 and 24 hours group (3 hours, 70.62 ± 1.36 mV, *n* = 16, compared to 48 hours, 66.47 ± 1.27 mV, *n* = 19, p = 0,0414, 24 hours, 70.04 ± 0.74 mV, *n* = 34 compared to 48 h, 66.47 ± 1.27 mV, *n* = 19, p = 0.0372). No statistically significant differences between the 3 hour and 24 hour groups were detected (3 hours, 70.62 ± 1.36 mV, *n* = 16, compared to 24 hours, 70.04 ± 0.74 mV, *n* = 34, p = 0.9198). Granule cells displayed APs upon stepwise depolarisation and no changes were detected in the amount of injected current needed to trigger an AP (Fig. 4B, Table 2) (n = 69, Kruskal-Wallis test, p = 0.5481). The number of AP for each depolarisation step (50 pA/step) at the three incubation time points is illustrated in Fig. 4D with example recordings of the lowest depolarisation step and the highest number of AP for each of the three time points. The input resistance and AP properties were recorded from each cell with no changes detected between the three groups (Mean and SEM in Table 2, individual cell values plotted against time and p-numbers in Supplementary Fig. S2).

**Table 2:** Intrinsic properties.

	3 h (*n* = 16)	24 h (*n* = 34)	48 h (*n* = 19)
RPM (mV)	−70.62 ± 1.36	−70.04 ± 0.74	−66.47 ± 1.27
Input R (MΩ)	185.50 ± 30.53	170.40 ± 60.83	164.7 ± 21.55
AP threshold (mV)	−43.05 ± 1.50	−43.82 ± 0.90	−42.87 ± 1.12
AP half-width (ms)	0.65 ± 0.03	0.63 ± 0.02	0.63 ± 0.03
AP amplitude (mV)	96.85 ± 3.04	99.27 ± 1.51	94.8 ± 2.01

### Spontaneous postsynaptic currents after 3, 24 and 48 hours of incubation

**Figure 5:**
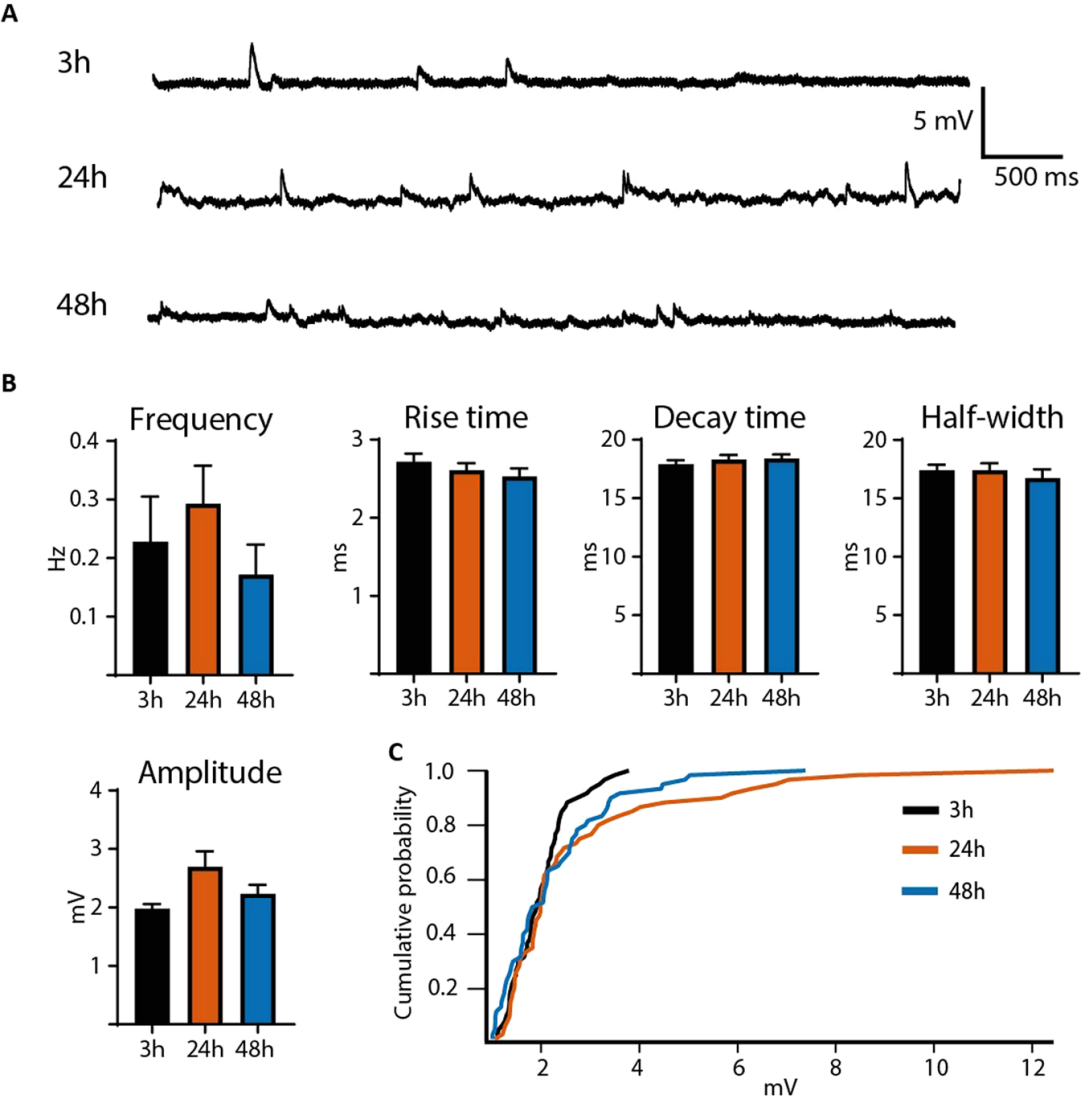
No difference in PSP properties was observed between 3, 24 and 48 hours of incubation. (**A**) Representative baseline recordings with normal aCSF showing EPSPs at 3, 24 and 48 hours of incubation. (**C**) No difference was detected when measuring frequency (3 h, *n* = 16, 24 h incubation, *n* = 34 and 48 h incubation, *n* = 19, Kruskal-Wallis test, p = 0.2307), amplitude (*n* = 60, Kruskal-Wallis test, p = 0.4391), half-width (*n* = 60, Kruskal-Wallis test, p = 0.9528), rise time (*n* = 60, Kruskal-Wallis test, p = 0.4583) or decay time (*n* = 60, Kruskal-Wallis test, p = 0.1921) of the PSPs generated by cells from slices incubated for 3, 24 and 48 hours. (**C**) PSP amplitude for each group (3, 24 and 48 hours) plotted as cumulative probability show no difference between the three groups (cumulative plot of PSP amplitude: comparing 3 and 24 hours incubation, n = 60 in each group, Kolmogorov-Smirnov test, p = 0.3752; comparing 3 and 48 hours incubation, n = 60 in each group, Kolmogorov-Smirnov test, p = 0.1813; comparing 24 and 48 hours incubation, n = 60 in each group, Kolmogorov-Smirnov test, p = 0.3752).

To investigate if the pattern of synaptic inputs to neurons was altered in slices over time in incubation, we recorded and analysed spontaneous PSPs in dentate granule neurons in slices at 3, 24 and 48 hours of incubation (Fig. 5, Table 3). The frequencies of PSP events sampled during the first 60 seconds of recording were not statistically different between the three groups (3 hours incubation: 0.23 ± 0.08 Hz n = 16, 24 hours incubation: 0.29 ± 0.06 Hz n = 34 and 48 hours incubation: 0.17 ± 0.05 Hz mV n = 19, Kruskal-Wallis test, p = 0.2307). No differences were detected in any parameter of PSPs analysed, including amplitude (3, 24 and 48 hours incubation, n = 60 in each group, Kruskal-Wallis test, p = 0.4391), half-width (3, 24 and 48 hours incubation, n = 60 in each group, Kruskal-Wallis test, p = 0.9528), rise time (3, 24 and 48 hours incubation, n = 60 in each group, Kruskal-Wallis test, p = 0.4583) or decay time (3, 24 and 48 hours incubation, n = 60 in each group, Kruskal-Wallis test, p = 0.1921). We also verified that no subtle change in PSP amplitude could be detected between the three groups by generating cumulative probability curves (Fig. 5C). No shift in the PSP amplitude cumulative probability curves could be detected (comparing 3 and 24 hours incubation, n = 60 in each group, Kolmogorov-Smirnov test, p = 0.3752; comparing 3 and 48 hours incubation, n = 60 in each group, Kolmogorov-Smirnov test, p = 0.1813; comparing 24 and 48 hours incubation, n = 60 in each group, Kolmogorov-Smirnov test, p = 0.3752), indicating maintained network connectivity over the entire time of incubation. Taken together, these data suggest that intrinsic electrophysiological properties and afferent synaptic inputs to dentate granule cells during the 48-hour incubation period are maintained without major alterations.

**Table 3:** Spontaneous synaptic potentials.

	3 h (*n* = 60)	24 h (*n* = 60)	48 h (*n* = 60)
Amplitude (mV)	1.98 ± 0.08	2.70 ± 0.26	2.23 ± 0.16
Half-width (ms)	17.41 ± 0.46	17.42 ± 0.57	16.75 ± 0.73
Rise time (ms)	2.72 ± 0.10	2.61 ± 0.09	2.53 ± 0.11
Decay time (ms)	17.91 ± 0.34	18.32 ± 0.38	18.43 ± 0.32

### Spontaneous and evoked epileptiform activity after 3, 24 and 48 hours of incubation

**Figure 6:**
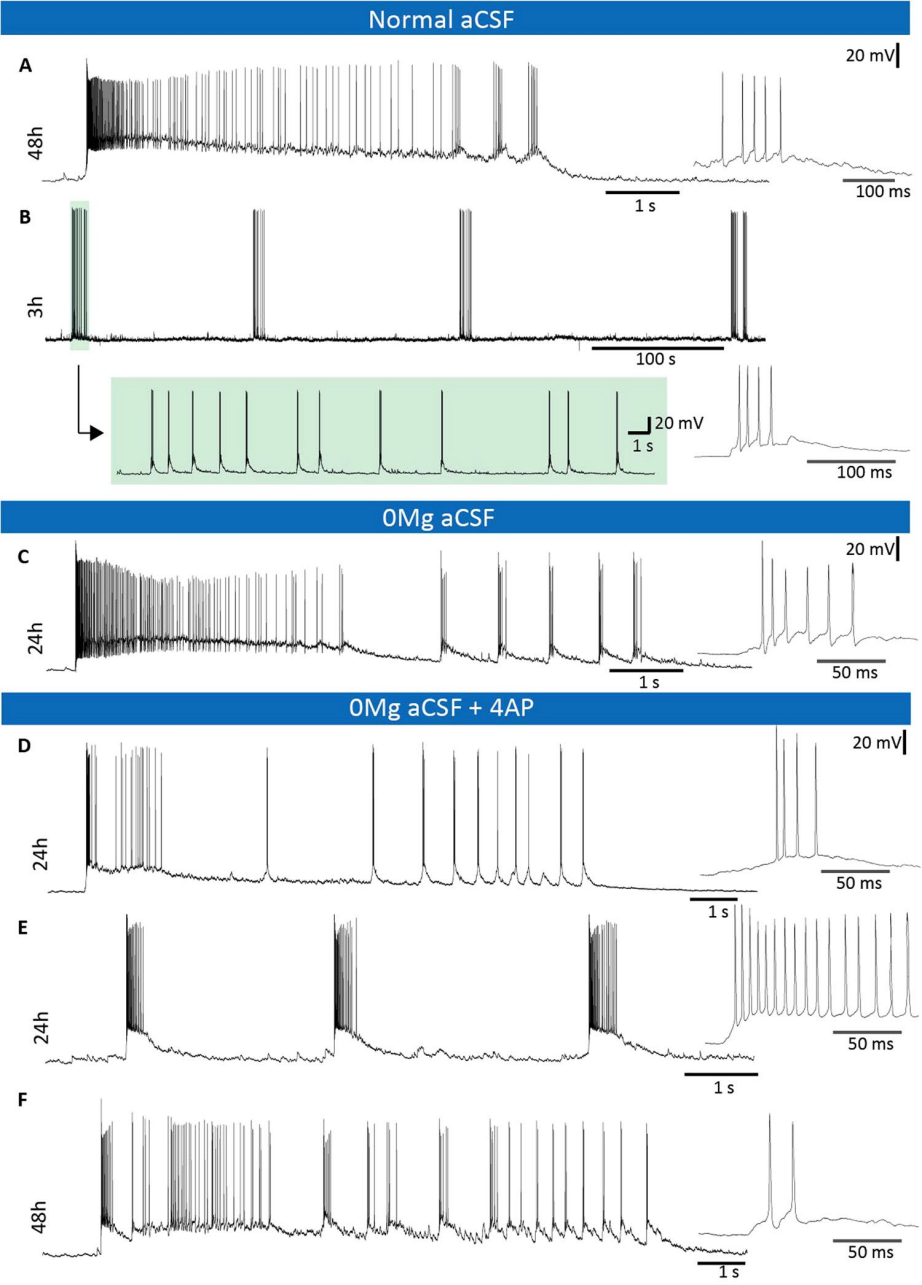
Spontaneous and evoked SLEs and epileptic burst activity observed after 3, 24 and 48 hours of incubation. (**A**) Spontaneous SLE was observed in normal aCSF after 48 hours of incubation, with a high frequency onset of 200 Hz and three terminating bursts. (**B**) Epileptiform burst activity recorded after 3 hours of incubation with 12–25 s long burst-trains every 3–11 min with 10–16 bursts in each train. The bursts consisted of up to five APs and each burst-train lasted 12–29 s. Between each burst the membrane potential returns to baseline until the onset of the next burst. (**C**) In slices incubated for 24 hours, SLEs with more than 150 Hz firing frequency at the onset and approximately 60 Hz in the following bursts were evoked by excluding Mg^2+^- from the aCSF. (**D**) By adding 4-AP to the [0Mg^2+^]-aCSF solution the activity became regular, with 150 Hz at the onset and around 40 Hz during the following bursts. (**E**) Example of regular bursts from a cell while perfused by the [0Mg^2+^]-aCSF solution with 4-AP added. (**F**) After 48 h of incubation it was still possible to evoke SLEs under perfusion of the [0Mg^2+^]-aCSF solution with 4-AP added.

To be able to use our platform as an *in vitro* validation tool for new antiepileptic treatments in human pharmacoresistant epileptic tissue, we investigated if dentate granule cells would display spontaneous or induced epileptiform activity at different time-points of incubation. We observed spontaneous epileptiform activity manifested as bursting AP activity in four dentate granule cells during perfusion with normal aCSF (Fig. 6A,B): three cells from one slice incubated for 3 hours, and one cell from a slice of a different patient incubated for 48 hours. Although the pattern of epileptiform activity of the cells from these two patients was somewhat different, the short burst-like epileptiform activity observed in the slice incubated for 3 hours was similar in all three cells (example, Fig. 6B). The epileptiform activity appeared in trains of AP bursts, each train lasting between 12 and 29 s, with the number of bursts during each train ranging from 10 to 16, and the number of APs in each burst from two to five APs. The frequency of the APs during one burst spanned from 43 Hz up to 294 Hz. The time interval between each burst train ranged from 3 to 11 min, one cell having more regular time interval between bursts (mean time of 6.00 ± 0.37 min). The spontaneous epileptiform activity (bursting) was abolished when NMDA (D-AP5) or AMPA (NBQX) receptor antagonist were applied to the perfusion medium (Supplementary Fig. S3). In the second patient tissue where spontaneous epileptiform activity was observed (Fig. 6A), the cell displayed one SLE with a high frequency AP onset (200 Hz), terminating with three distinct bursts.

The low rate and irregularity of spontaneous epileptiform events in normal aCSF makes this approach inadequate to evaluate and validate a potential therapeutic effect of novel treatment strategies. Therefore, to evoke more regular and higher frequency epileptiform events, we tested 3 different protocols that have been previously used for this purpose: (i) [0Mg^2+^]-aCSF alone, or in combination with (ii) electrical stimulation or (iii) potassium channel blocker 4-AP. Reducing magnesium-block of the NMDA receptor by [0Mg^2+^]-aCSF has been shown to readily induce seizure-like activity in human hippocampal slices^20^. In our study, application of [0Mg^2+^]-aCSF gave rise to SLEs in three out of six recorded dentate granule cells from 6 slices (Fig. 5C), with a high frequency onset of APs of more than 150 Hz, followed by sporadic bursts with AP frequency of more than 50 Hz. Combining [0Mg^2+^]-aCSF with electrical stimulation, similar to stimulus-induced bursting (STIB), induced SLEs with similar burst frequency (in 2 dentate granule cells from 2 slices, Supplementary Fig. S4). Finally, with the addition of the potassium channel blocker 4-AP to the [0Mg^2+^]-aCSF, we observed robust and regular bursting in 6 out of 11 dentate granule cells recorded (example Fig. 5D–F). The time interval between the bursts ranged from about 1 s to 41 s, with burst duration ranging from 16 to 630 ms. Number of APs during a burst varied from 2 (the minimum number of APs to be defined as a burst) to a maximum of 36, with an average of 6.9 APs per burst. Firing frequency of APs during the bursts was on average 54 Hz, ranging between 8 Hz and 264 Hz. In one cell, apart from the bursts, a SLEs with high frequency AP onset (180 Hz) and with 3 to 10 terminating bursts was also observed. Another cell displayed bursting of APs in trains with a duration between 10 and 13 s. The number of bursts during each train ranged from 16 to 22, and the number of APs in each burst ranged from 2 to 17. The first burst in each train displayed similar characteristics as an SLE, with a high frequency onset and longer duration, but was lacking the terminating bursts. The evoked epileptiform activity was reduced in the presence of the NMDA receptor antagonist D-AP5, further decreased by adding AMPA receptor antagonist NBQX, and was fully abolished in the presence of NMDA, AMPA and GABA (PTX) receptor antagonists (See supplementary Fig. S5). This suggests that the epileptiform activity evoked by [0Mg^2+^]/4-AP-aCSF is dependent on both glutamatergic and GABAergic receptors. Further, simultaneous paired field and whole-cell recordings from the molecular layer and a granule cell showed recurrent, epileptiform activity in field recordings, synchronised to burst activity in the whole-cell recording (See supplementary Fig. S6). Applying our incubation protocol to human resected tissue from a cortical dysplasia patient demonstrated both viable cells and normal electrophysiological properties at 24 hours time point, with an average RMP of −69.2 ± 3.1 mV, and AP amplitude 93.4 ± 6.7 mV (n = 5), as well as robust rhythmic epileptiform bursts in response to [0Mg^2+^]/4-AP-aCSF (See supplementary Fig. S7), indicating that maintained neuronal properties over the first 24-hours is not dependent on the selective resilience of DG neurons to stress, but is rather due to the used incubation procedure. Further studies are needed, however, to confirm viability of cortical neurons over 48-hours.

## Discussion

Here we present a method for human brain slice incubation where human hippocampal slices are viable and functionally preserved for 48 hours after resection from patients with drug-resistant temporal lobe epilepsy. We provide evidence that this unique tissue could be used for electrophysiological experiments for a prolonged period, therefore enabling increased number of experiments to be carried out from the same resection surgery, as well as allowing for broader experimental approaches, such as fast-expressing viral vector based gene therapy strategy validations. Moreover, we present data demonstrating that the dual flow-recording chamber represents an unprecedented opportunity for visually guided whole-cell patch-clamp recording of individual neurons, and at the same time enabling induction of seizure-like activity in human hippocampal tissue slices. We also report that whole-cell recordings from human dentate granule cells reveal robust seizure-like activity in epileptic dentate gyrus up to 48 hours of incubation. Interestingly, *spontaneous* epileptiform activity in the epileptic dentate gyrus, an area where no such activity has been previously reported, was occasionally observed in normal aCSF. The presented method significantly expands the time span for recordings in acute human brain slices, substantially increasing the window of opportunity for electrophysiological recordings, and thereby maximising the period for broad electrophysiological data acquisition when using this unique human brain tissue.

We found that incubating human hippocampal slices under interface conditions for 48 hours did not lead to a decrease in number of neurons in the dentate granule layer (Fig. 2, Supplementary Table 2), nor did it reduce the number of NPY-expressing interneurons (Fig. 3, Supplementary Table 2), suggesting good survival capacity of the slices under these conditions. We detected, however, an increase in the number of activated microglia after 48 hours (Supplementary Table 2). When activated, microglial cells start expressing pro-inflammatory cytokines, such as interleukins, TNF-α and IFN-γ, to recruit neighbouring microglia and trigger broader inflammatory processes around them^21^. Activated microglia are also known for their ability to modulate neuronal circuitry in their vicinity, either by removing presynaptic terminals (“synaptic stripping”^22,23^) or regulating synaptic receptors through signalling molecules^23^, for example by up-regulation of postsynaptic AMPA-receptor expression through signalling via TNF-α^22^. Present data in human slices, however, do not indicate that such changes occur in synaptic transmission, at least in the dentate gyrus, since our electrophysiological experiments show no significant changes in PSP-properties at 48 h: neither amplitude, duration, rise time nor decay time of PSPs were altered. No significant differences were detected in the frequency of spontaneous postsynaptic events, indicating that synaptic connectivity, as measured by PSPs, did not change over the 48 hours of incubation. The RMP of dentate granule cells was slightly lower at this time-point (for about 3 mV on average) although other intrinsic membrane properties or AP firing properties were not different between 3, 24 and 48 hours of incubation (Fig. 4, Table 2), indicating that the cells have functionally intact membrane, with ion pumps and channels, capable of maintaining RMP and generation of normal APs. RMP values for human dentate gyrus cells has previously reported to be -62.9 ± 1.24 mV^24^, which is in agreement with our data.

Spontaneous rhythmic burst activity with clear paroxysmal depolarisation shifts (PDS) was observed, in normal aCSF, in four dentate granule cells (three with AP during the PDS and one without the AP) in one slice after 3 hours of interface incubation. To our knowledge this is the first time spontaneous epileptiform burst activity has been recorded in the dentate gyrus of human epileptic hippocampal slices^25,26^. Previously, Richard Miles’ group showed spontaneous activity, recorded in the subiculum and the CA2 area of the human hippocampus^13,27^. The burst activity recorded in the dentate gyrus by us is very similar in shape and frequency to the activity previously recorded from pyramidal cells in the subiculum^13^. The major difference is that this activity seen in the dentate gyrus came in burst-trains with several minutes of silence in between the bursts, while previously observed activity from subiculum was continuous rhythmic burst activity without long intervals. The common consensus on describing characteristics for SLEs is a fast onset followed by irregular spiking, ending with periodic bursting^28^. This matches with our spontaneous recordings: an onset of AP frequency over 150 Hz followed by attenuated AP frequency that stabilizes for a few seconds and then terminates by three clearly defined bursts. In general, SLE activity (assessed by field recordings in temporal neocortex) has been demonstrated previously exclusively in the interface recording chamber^14^, but has not been observed using the submerged-recording chamber as shown here. Our findings support the conclusion that the submerged dual-flow perfusion chamber presumably allows for comparable conditions, e.g., oxygen levels, to the interface-recording chamber, without compromising visual access for whole-cell patch-clamp recordings as is the case for the interface-recording chamber.

Epileptiform activity in the dentate gyrus (as judged by whole-cell patch-clamp recordings from granule cells) was successfully evoked by both [0Mg^2+^]-aCSF as well as [0Mg^2+^]/4-AP-aCSF. In cells perfused with [0Mg^2+^]-aCSF, we observed SLEs with similar features to the spontaneous SLE (Fig. 5). The recurrence of these SLEs was highly variable between the recorded cells, ranging from a single SLE to one every 8 minutes. The slices perfused with [0Mg^2+^]/4-AP-aCSF rarely displayed SLEs (1 in 10 cells), but instead exhibited strong AP bursts in 5 out of 10 cells (Fig. 5D–F). The epileptiform activity evoked by [0Mg^2+^]/4-AP-aCSF were comparable to the spontaneous bursts in aCSF. It is clear that both [0Mg^2+^]-aCSF and [0Mg^2+^]/4-AP-aCSF can generate epileptiform activity as revealed by recordings obtained from the dentate granule cells in the double-flow chamber. The [0Mg^2+^]-aCSF caused SLEs very similar to the one that occurred spontaneously during normal aCSF perfusion.

To be able to test new compounds and treatments, however, robust and continuous epileptiform activity is required. The slices perfused with [0Mg^2+^]/4-AP-aCSF generate such robust activity, with bursts that exhibit similarities to those that follow spontaneous SLEs seen under perfusion with normal aCSF, making this protocol suitable for evaluating novel treatments for suppression of seizure-like events in human epileptic tissue.

Taken together, the method presented here with long incubation time and ability to generate robust epileptiform activity is a favourable platform for pre-screening of novel therapeutic approaches, particularly those that require longer observation times (e.g. to allow for viral vector-based gene expression), as a validation step in patient-derived pharmacoresistant epileptic tissue before proceeding to clinical trials. In addition, the double-flow chamber makes it possible to induce seizure-like events with a visual approach for whole-cell recordings readily available. The latter may increase the sensitivity of the platform, as well as deepen our understanding of how individual neurons contribute to epileptiform activity in human pharmacoresistant epileptic tissue.

## Electronic supplementary material

Supplementary Information
