# Supplementary material for: Prolonged life of human acute hippocampal slices from temporal lobe epilepsy surgery
Source: Sci Rep. 2018 Mar 7;8:4158. doi: 10.1038/s41598-018-22554-9 (PMC5841387; doi:10.1038/s41598-018-22554-9)
Supplement: Supplementary file 1 — Supplementary Information [file 41598_2018_22554_MOESM1_ESM.pdf]

Supplementary Figures and Tables

Prolonged life of human acute hippocampal slices from temporal lobe epilepsy surgery

Wickham J[1], G Brödjegård N[1], Vighagen R[1], Pinborg LH[2], Bengzon J[3,4], Woldbye DPD[5], Kokaia M[1] and Andersson M[1]

Affiliation. [1]Epilepsy Centre, Department of Clinical Sciences, Lund University, Sölvegatan 17, 223 62 Lund, Sweden. [2] Epilepsy Clinic and Neurobiology Research Unit, Department of Neurology, Copenhagen University Hospital, Rigshospitalet, Building 6931, Blegdamsvej 9, DK-2100, Copenhagen, Denmark. [3]Department of Clinical Sciences, Lund University, Skåne University Hospital Lund, S-22185 Lund, Sweden. [4]Lund Stem Cell Center, BMC B10, Klinikgatan 26, 221 84 Lund, Sweden [5]Laboratory of Neuroplasticity, Center for Neuroscience, University of Copenhagen, Copenhagen, Denmark.

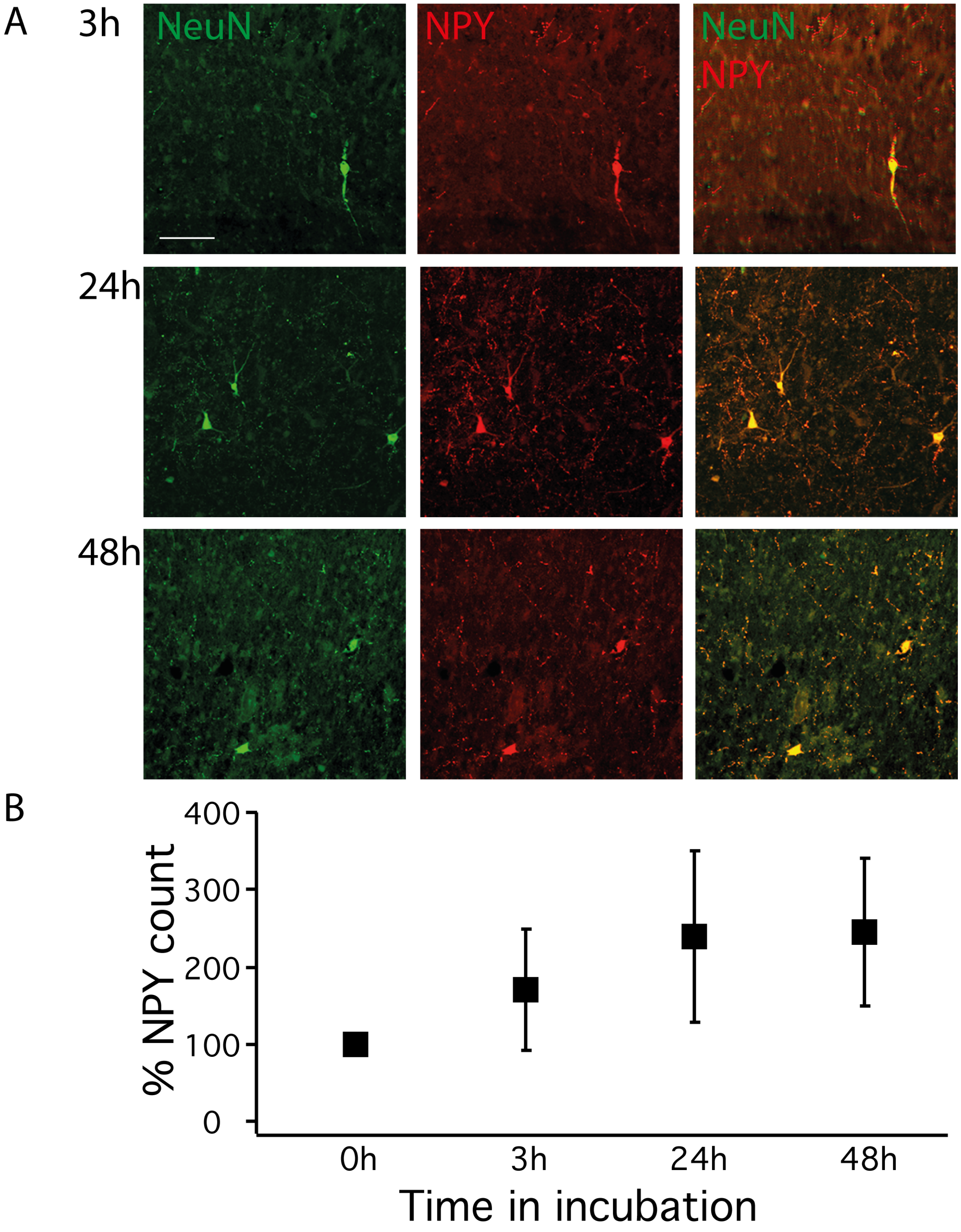

Supplementary Figure 1. No change in NPY-containing hilar interneuron number during time in incubation. An Example immunostainings from the same patient (number 14) in brain slices incubated for 3, 24 or 48 h**.** Scale bar 100 um. B, Average of NPY-containing neurons/mm^2 normalized to 0h (0h: 4 patients, 24h: 3 patients and 48h: 4 patients).

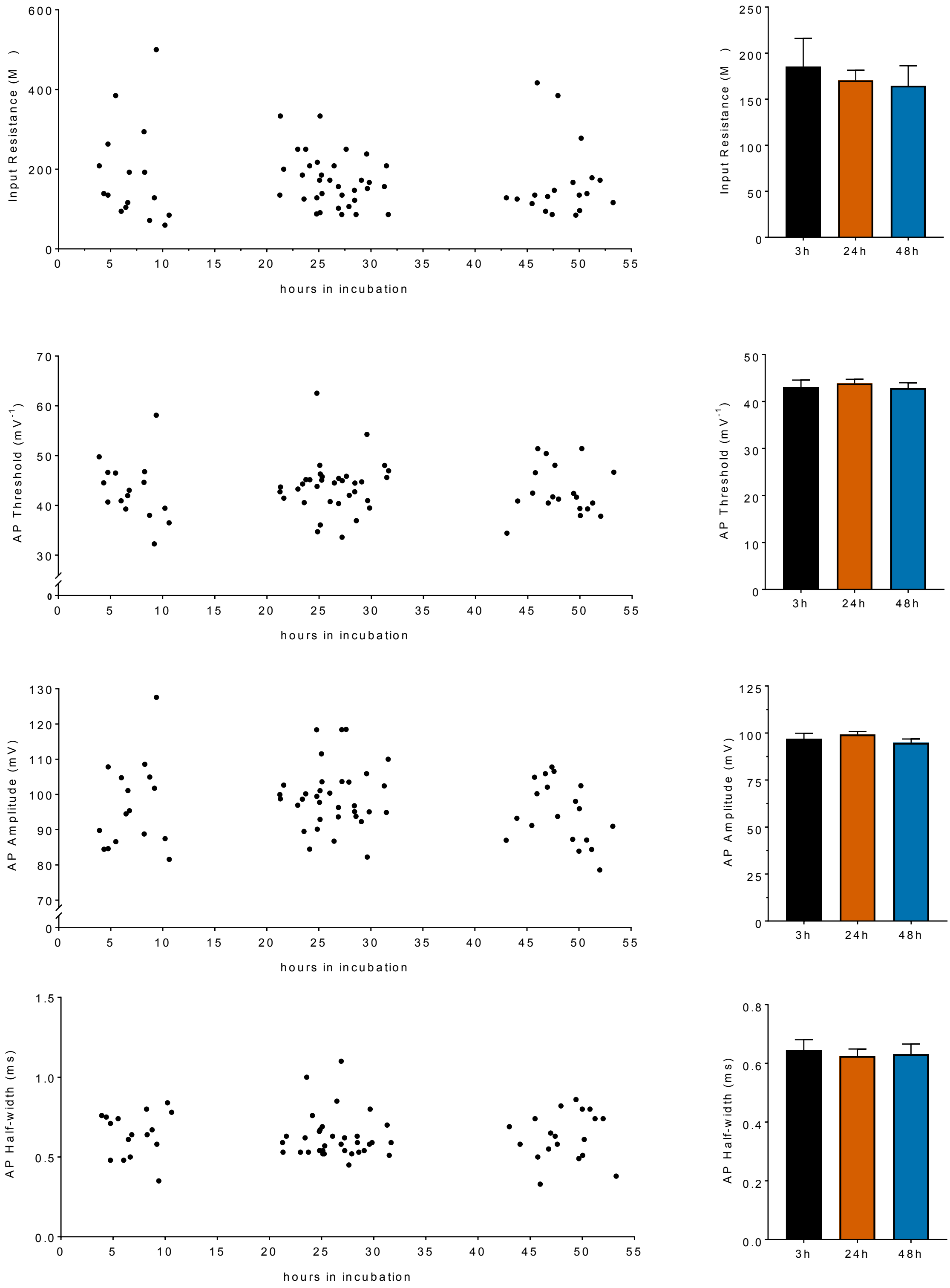

Supplementary Figure 2. Intrinsic properties of granule cells are stable over incubation time

No changes can be detected in input resistance or AP properties between the three slice incubation time points of 3, 24 and 48 hours (Average and SEM values for each group for

respective parameters can be found in table 2). (A) To the left, input resistance values for each individual cells are plotted against the actual time for incubation (not binned), to the right the average and SEM from the three groups 3, 24 and 48 hours (binned) show no difference between the three groups (3, 24 and 48 hours of incubation, n = 16, 34 and 19 respectively, Kruskal-Wallis test, p = 0.5691). (B) To the left AP threshold for each individual cells is plotted against the actual time in incubation (not binned), while to the right the average and SEM (binned) from the three groups 3, 24 and 48 hours show no difference between the groups (3, 24 and 48 hours of incubation, n = 16, 34 and 19 respectively, Kruskal-Wallis test, p = 0.6304). (C) To the left AP amplitude for each individual cells is plotted against the actual time in incubation (not binned), to the right the average and SEM from the three groups 3, 24 and 48 hours (binned) show no difference between the groups (3, 24 and 48 hours of incubation, n = 16, 34 and 19 respectively, Kruskal-Wallis test, p = 0.3424). (D) To the left AP half-width for each individual cells is plotted against the actual time in incubation (not binned), to the right the average and SEM (binned) from the three groups 3, 24 and 48 hours show no difference between the groups (3, 24 and 48 hours of incubation, n = 16, 34 and 19 respectively, Kruskal-Wallis test, p = 0.5849).

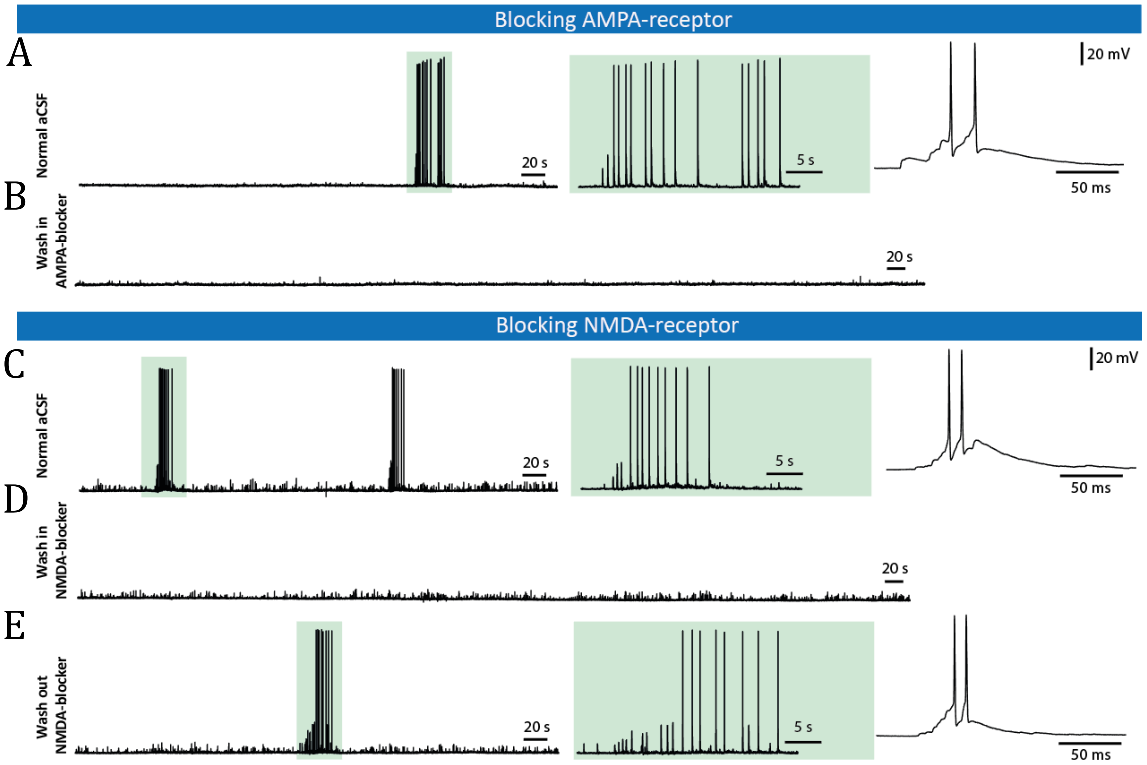

Supplementary Figure 3. Abolishing spontaneous epileptiform activity by blocking NMDA or AMPA receptors.

(A) Spontaneous epileptiform activity was observed as burst trains in normal aCSF. (B) After applying AMPA receptor antagonist NBQX to the perfusion medium, burst trains were abolished . (C) In another cell, spontaneous epileptiform activity of burst trains (D) was abolished by NMDA receptor antagonist D-APV application. (E) After wash-out of D-APV, burst trains reappeared.

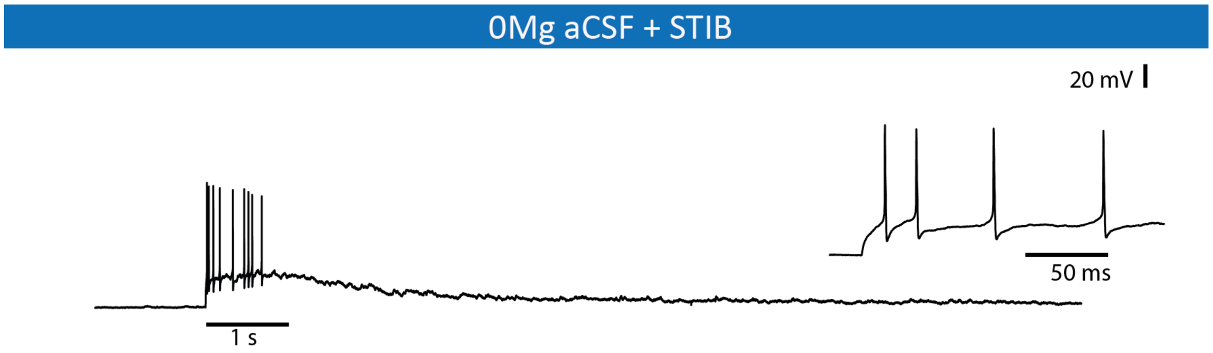

Supplementary Figure 4. Epileptiform activity evoked by [0Mg2+]-aCSF and electrical stimulations

Combining [0Mg2+]-aCSF and electrical stimulations resulted in a few non-recurrent bursts.

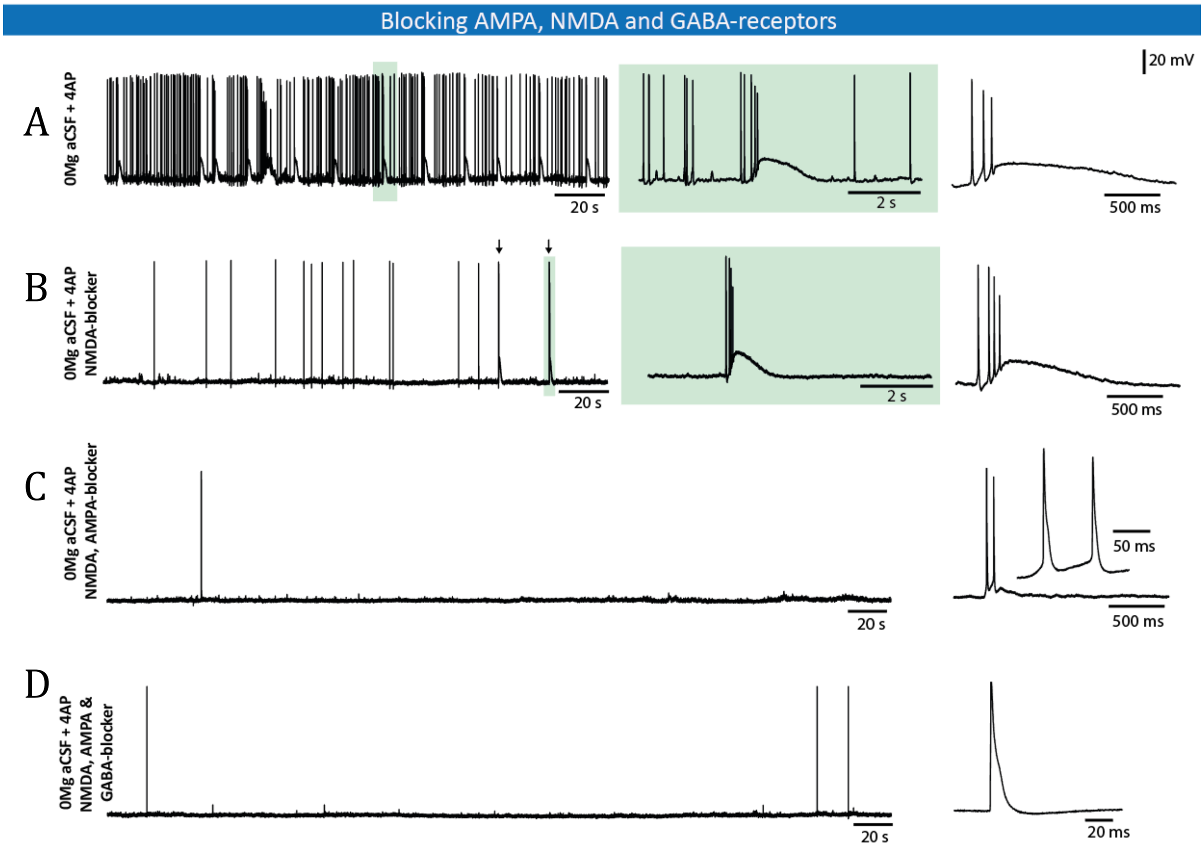

Supplementary Figure 5. Abolishing evoked epileptiform activity by blocking NMDA, AMPA and GABA receptors

(A) Epileptiform burst activity evoked by [0Mg^{2+}]/4AP-aCSF recorded from a granule cells of a slice incubated for 48h. The green area highlights one of the bursts with faster timescale. (B) The bursting activity is reduced when the NMDA receptor antagonist D-APV is applied, arrows indicating the only two bursts in the recording. (C) When the AMPA receptor antagonist NBQX was added only one short burst was recorded from the cell, while after application of the GABA receptor antagonist PTX (D) only a few single APs were present and the burst activity was completely abolished.

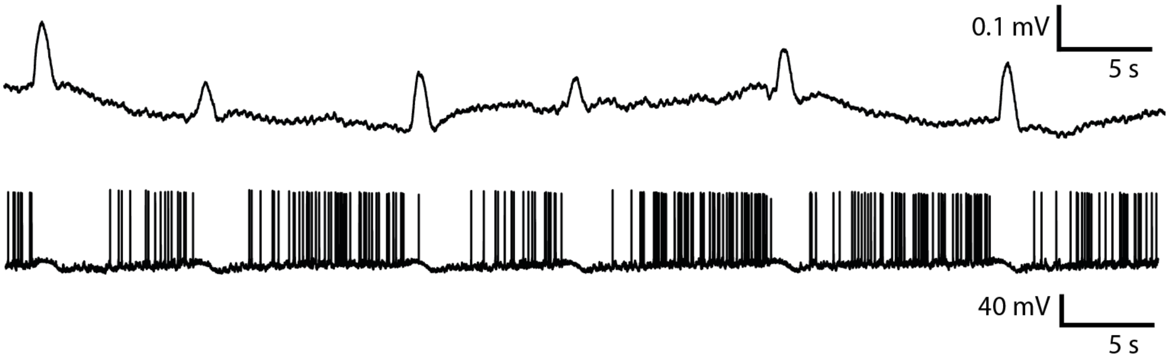

Supplementary Figure 6. Epileptiform activity evoked by [0Mg2+]/4AP-aCSF recorded from a dentate granular cell and field simultaneously

Field recordings from the molecular layer of the dentate gyrus display recurrent epileptiform waves that are synchronised with burst-hyperpolarizing sequence recorded simultaneously in a dentate granule cell.

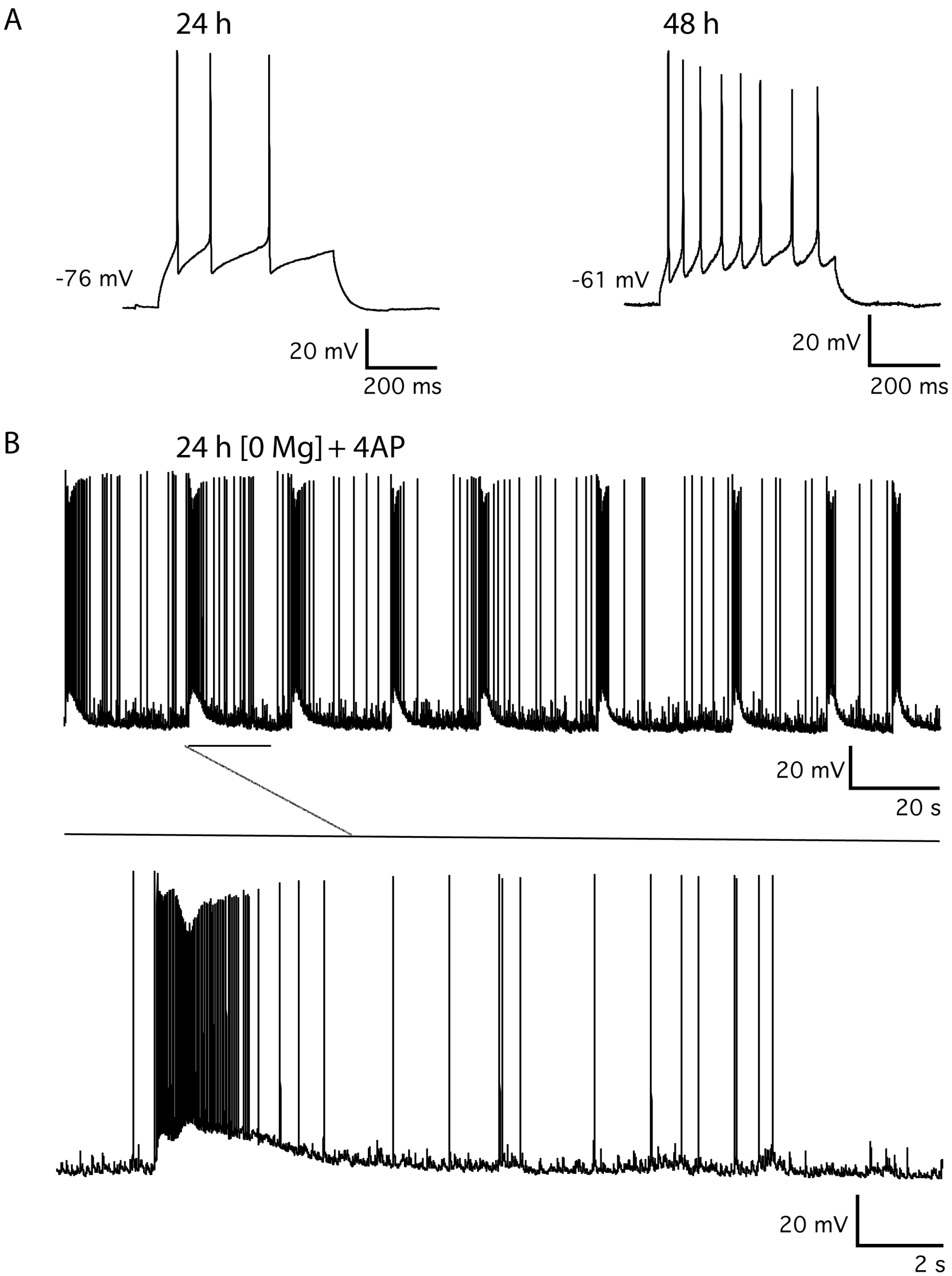

Supplementary Figure 7. Recordings from human cortical slices after 24 and 48 hours of incubation. A. Example sweeps of neurons recorded after 24 or 48 hours of incubation in slices from the same patient tissue, from a patient diagnosed with Focal Cortical dysplasia type II. Neurons respond with action potentials to current injection. B, Example sweep from a cortical neuron after 24 hours of slice incubation show robust

bursting activity in $0Mg^{2+}$ and 4-AP (100 um) aCSF, with a regular occurring burst pattern (magnified in the lower panel).

Supplementary Table 1. Antibodies

Primary Ab	Raised in	Dilution	*Provider*	Secondary	*Provider*
**NeuN**	Rabbit	1:500	*Abcam (ab104225)*	Alexa Fluor 488	*Jackson ImmunoResearch (#711-545-152)*
**Cas3**	Rabbit	1:200	*Abcam (ab2302)*	Cy3	*Jackson ImmunoResearch (#711-165-152)*
**NPY**	Rabbit	1:500	*Sigma (N9528)*	Cy3	*Jackson ImmunoResearch (#711-165-152)*
**GFAP**	Mouse	1:500	*Sigma (G3893)*	Alexa Fluor 488	*Jackson ImmunoResearch (#715-545-150)*
**Iba1**	Rabbit	1:500	*WAKO (#019-19741)*	Cy3	*Jackson ImmunoResearch (#711-165-152)*

Supplementary Table 2. Immunohistochemical quantification

Patient 1

	0h (mean±SD)	24h (mean±SD)
**NeuN+CASP3**		
NeuN/mm2	89,6025 ± 3,3109	152,5125 ± 10,3863
CASP3/mm2	3,7567 ± 0,2416	8,8689 ± 2,2070
CASP3/NeuN	0,0420 ± 0,0041	0,0577 ± 0,0117
**NPY + NeuN**		
NPY/mm2	0,1821 ± 0,0273	0,0821 ± 0,0182
**Iba1**		
Ramified (part of total n)	0,8022 ± 0,0455	0,0128 ± 0,0177
Intermediate (part of total n)	0,1935 ± 0,0405	0,7358 ± 0,1235
Activated (part of total n)	0,0043 ± 0,0086	0,2514 ± 0,1253
Iba1/mm2	16,7397 ± 2,8295	17,9780 ± 1,8061
**GFAP**		
Mean Grey Value	14,4695 ± 4,2334	21,903 ± 6,3447
Min Grey Value	3,25 ± 1,2583	6 ± 2,1602
Max Grey Value	48 ± 4,8305	64,25 ± 5,3151
Integrated Optical Density	14469452 ± 4233209	21902929 ± 6344678
Corrected Total Fluorescence	10838702 ± 3672283	16875679 ± 4846690

Patient 2

	0h (mean±SD)	3h (mean±SD)	24h (mean±SD)	48h (mean±SD)
**NeuN+CASP3**				
NeuN/mm2	78,0309 ± 20,2153	95,8512 ± 15,1702	133,8846 ± 16,3634	118,8438 ± 11,4896
CASP3/mm2	5,1616 ± 2,6947	3,5644 ± 1,5470	37,9356 ± 9,5290	13,6988 ± 1,4512
CASP3/NeuN	0,0628 ± 0,0190	0,0384 ± 0,0209	0,2807 ± 0,0415	0,1167 ± 0,0209
**NPY + NeuN**				
NPY/mm2	0,1170 ± 0,0348	0,0956 ± 0,0545	0,1733 ± 0,0483	0,1011 ± 0,0249
**Iba1**				
Ramified (part of total n)	0,6857 ± 0,0795	0,6645 ± 0,0503	0,1555 ± 0,0271	0,0212 ± 0,0235
Intermediate (part of total n)	0,3101 ± 0,0782	0,3295 ± 0,0396	0,6587 ± 0,0844	0,3390 ± 0,0452
Activated (part of total n)	0,0042 ± 0,0085	0,0060 ± 0,0119	0,1858 ± 0,0638	0,6409 ± 0,0602
Iba1/mm2	20,0916 ± 3,2206	14,8407 ± 1,1568	15,1987 ± 4,2766	12,2121 ± 3,3140
**GFAP**				
Mean Grey Value	3,0025 ± 1,5489	3,2898 ± 1,7839	3,3668 ± 1,3488	4,7368 ± 2,4647

Min Grey Value	1 ± 0,8165	1 ± 0,8165	0,5 ± 0,5774	1,25 ± 0,5
Max Grey Value	17,5 ± 3,6968	20 ± 1,4142	16,75 ± 6,8981	19,75 ± 6,5
Integrated Optical Density	3002570,371 ± 1548683,659	3289696,297 ± 1784036,208	3366888,272 ± 1349011,812	4736642,902 ± 2464396,814
Corrected Total Fluorescence	1723820,371 ± 390026,7633	1745446,297 ± 570866,666	1543388,272 ± 514589,9606	2610642,902 ± 1400450,784

Patient 3

	0h (mean±SD)	3h (mean±SD)	24h (mean±SD)	48h (mean±SD)
**NeuN+CASP3**				
NeuN/mm2	102,4970 ± 11,2607	103,6369 ± 18,8157	126,7554 ± 13,0993	153,6422 ± 9,9249
CASP3/mm2	10,4420 ± 3,1706	8,8764 ± 3,6337	18,5323 ± 1,3005	14,3525 ± 2,3525
CASP3/NeuN	0,1016 ± 0,0269	0,0829 ± 0,0221	0,1466 ± 0,0057	0,0931 ± 0,0117
**NPY + NeuN**				
NPY/mm2	0,0966 ± 0,0222	0,0990 ± 0,0347	0,2014 ± 0,0430	0,2239 ± 0,0741
**Iba1**				
Ramified (part of total n)	0,7403 ± 0,0529	0,5889 ± 0,0045	0,1123 ± 0,0180	0,0426 ± 0,0321
Intermediate (part of total n)	0,2389 ± 0,0494	0,3871 ± 0,0326	0,7839 ± 0,0601	0,3142 ± 0,0644
Activated (part of total n)	0,0208 ± 0,0315	0,0240 ± 0,0326	0,1038 ± 0,0604	0,6431 ± 0,0918
Iba1/mm2	19,4072 ± 0,9051	15,9378 ± 2,7591	13,6946 ± 1,4200	18,9017 ± 3,3424
**GFAP**				
Mean Grey Value	6,0343 ± 2,2426	5,7598 ± 2,7459	4,9630 ± 2,2873	4,4578 ± 0,9780
Min Grey Value	1,75 ± 0,5	1,75 ± 0,9574	1,5 ± 0,5774	1 ± 0
Max Grey Value	40,25 ± 3,4034	47,5 ± 7,3711	40 ± 3,8297	48,75 ± 6,2383
Integrated Optical Density	6034299,692 ± 2242573,163	5760008,642 ± 2746017,655	4962900,926 ± 2287307,986	4457829,012 ± 978016,9915
Corrected Total Fluorescence	3064299,692 ± 1570785,603	2946008,642 ± 1583169,743	2474650,926 ± 1120801,533	2858329,012 ± 641258,6406

Patient 4

	0h (mean±SD)	3h (mean±SD)	24h (mean±SD)	48h (mean±SD)
**NeuN+CASP3**				
NeuN/mm2	269,362 ± 15,702	283,555 ± 7,701	284,898 ± 18,695	276,220 ± 27,993
CASP3/mm2	19,165 ± 4,105	30,537 ± 11,717	29,0439 ± 13,348	21,308 ± 4,796
CASP3/NeuN	0,0711 ±0,0140	0,108 ± 0,0425	0,1 ± 0,0399	0,0767 ± 0,0111
**NPY + NeuN**				
NPY/mm2	0,26384 ± 0,0756	0,4258 ± 0,096	0,489 ± 0,0496	0,325 ± 0,116
**Iba1**				

Ramified (part of total n)	0,7229 ± 0,04669	0,6771 ± 0,0405	0,0931 ± 0,0281	0,0452 ± 0,0322
Intermediate (part of total n)	0,2738 ± 0,0401	0,2955 ± 0,0279	0,8151 ± 0,0965	0,3686 ± 0,0225
Activated (part of total n)	0,00333 ± 0,00667	0,02737 ± 0,02195	0,0918 ± 0,07349	0,5861 ± 0,0518
Iba1/mm2	26,112 ± 1,652	22,372 ± 7,489	13,773 ± 4,249	13,608 ± 2,853
**GFAP**				
Mean Grey Value	12,277 ± 3,5612	14,1798 ± 8,361	15,375 ± 6,805	9,656 ± 5,6399
Min Grey Value	3,75 ± 0,9574	4,25 ± 2,0616	4,25 ± 2,0616	2,5 ± 0,58
Max Grey Value	49,75 ± 3,304	48,25 ± 10,436	59 ± 18,89	43,5 ± 4,7258
Integrated Optical Density	12276490 ± 3561308	14179769 ± 8360652	15374764 ± 6804925	9656189 ± 3829977
Corrected Total Fluorescence	7120990 ±2133528	8634269 ± 6048047	9266014 ± 4752328	5240689 ± 3829977
